# Supplementary material for: Genomic insights into the taxonomic status and bioactive gene cluster profiling of Bacillus velezensis RVMD2 isolated from desert rock varnish in Ma’an, Jordan
Source: PLoS One. 2025 Apr 24;20(4):e0319345. doi: 10.1371/journal.pone.0319345 (PMC12021177; doi:10.1371/journal.pone.0319345)
Supplement: S1 Table — The classification is inferred by the maximum Average Amino Acid Identity (AAI) found against all genomes in the database. The p-value, estimated from the empirical distribution observed in all NCBI RefSeq reference genomes, indicates the probability of a different classification with the observed AAI. Closest relatives identified were Bacillus velezensis NZ CP036527 (99.86% ANI) and Bacillus velezensis NZ CP010556 (99.35% ANI). Significance at p-value below: ***0.05, ****0.01. (DOCX) [file pone.0319345.s001.docx]

**S1 Table.** Taxonomic classification and significance levels for the query dataset, analyzed through MiGA (Microbial Genome Atlas)(Rodriguez et al., 2018). The classification is inferred by the maximum Average Amino Acid Identity (AAI) found against all genomes in the database. The *p*-value, estimated from the empirical distribution observed in all NCBI RefSeq reference genomes, indicates the probability of a different classification with the observed AAI. Closest relatives identified were *Bacillus velezensis* NZ CP036527 (99.86% ANI) and *Bacillus velezensis* NZ CP010556 (99.35% ANI).

| Taxonomic Classification | | *p-value* |
| --- | --- | --- |
| Domain | Bacteria | 0**** |
| Phylum | Firmicutes | 0**** |
| Class | *Bacilli* | 0**** |
| Order | *Bacillales* | 0.000129**** |
| Family | *Bacillaceae* | 0.000194**** |
| Genus | *Bacillus* | 0.00116**** |
| Species | *Bacillus velezensis* | 0.00433**** |

Significance at *p*-value below: ***0.05, ****0.01.

**REFERNCES**

Rodriguez, R. L., Gunturu, S., Harvey, W. T., Rosselló-Mora, R., Tiedje, J. M., Cole, J. R., & Konstantinidis, K. T. (2018). The Microbial Genomes Atlas (MiGA) webserver: taxonomic and gene diversity analysis of Archaea and Bacteria at the whole genome level. *Nucleic Acids Res, 46*(W1), W282-w288. doi:10.1093/nar/gky467
