# Supplementary material for: Genomic insights into the taxonomic status and bioactive gene cluster profiling of Bacillus velezensis RVMD2 isolated from desert rock varnish in Ma’an, Jordan
Source: PLoS One. 2025 Apr 24;20(4):e0319345. doi: 10.1371/journal.pone.0319345 (PMC12021177; doi:10.1371/journal.pone.0319345)
Supplement: S2 Table — Displays the pairwise digital DNA-DNA hybridization (dDDH) values, including confidence intervals (C.I.), between RVMD2 genome and selected type-strain genomes using GBDP formula d4 (GGDC formula 2), which sums all identities found in high-scoring pairs (HSPs) divided by the overall HSP length. (DOCX) [file pone.0319345.s002.docx]

**S2 Table. Pairwise dDDH values between *Bacillus velezensis* strain RVMD2 genome and Type-Strain Genomes**. Displays the pairwise digital DNA-DNA hybridization (dDDH) values, including confidence intervals (C.I.), between RVMD2 genome and selected type-strain genomes using GBDP formula *d4* (GGDC formula 2), which sums all identities found in high-scoring pairs (HSPs) divided by the overall HSP length.

| Query strain | Subject strain | dDDH (d4, in %) | C.I. (d4, in %) | G+C content difference (in %) |
| --- | --- | --- | --- | --- |
| *Bacillus velezensis* strain RVMD2 | *Bacillus velezensis* NRRL B-41580 | 80.2 | [77.2 - 82.8] | 0.37 |
|  | *Bacillus amyloliquefaciens* subsp. *plantarum* FZB42 | 80 | [77.1 - 82.6] | 0.53 |
|  | *Bacillus methylotrophicus* KACC 13105 | 79.5 | [76.5 - 82.1] | 0.49 |
|  | *Bacillus siamensis* KCTC 13613 | 56.7 | [54.0 - 59.5] | 0.39 |
|  | *Bacillus vanillea* XY18 | 56.7 | [54.0 - 59.5] | 0.38 |
|  | *Bacillus amyloliquefaciens* DSM 7 | 55 | [52.3 - 57.7] | 0.14 |
|  | *Bacillus nakamurai* NRRL B-41091 | 30.8 | [28.4 - 33.3] | 0.68 |
|  | *Bacillus cabrialesii* subsp. *tritici* TSO2 | 20.7 | [18.5 - 23.1] | 1.93 |
|  | *Bacillus subtilis* ATCC 6051 | 20.5 | [18.2 - 22.9] | 2.43 |
|  | *Bacillus cabrialesii* TE3 | 20.4 | [18.2 - 22.8] | 1.83 |
|  | *Bacillus rugosus* SPB7 | 20.4 | [18.1 - 22.8] | 2.82 |
|  | *Bacillus mojavensi*s KCTC 3706 | 20.3 | [18.0 - 22.7] | 2.28 |
|  | *Bacillus vallismortis* DV1-F-3 | 20.1 | [17.9 - 22.5] | 2.18 |
