# Supplementary material for: Genomic insights into the taxonomic status and bioactive gene cluster profiling of Bacillus velezensis RVMD2 isolated from desert rock varnish in Ma’an, Jordan
Source: PLoS One. 2025 Apr 24;20(4):e0319345. doi: 10.1371/journal.pone.0319345 (PMC12021177; doi:10.1371/journal.pone.0319345)
Supplement: S3 Table — (DOCX) [file pone.0319345.s003.docx]

**S3 Table.** Distribution of Carbohydrate-Active Enzymes (CAZys) in the *Bacillus velezensis* RVMD2 genome as analyzed by Protologger v0.99 [1].

| Category | Family | Occurrences |
| --- | --- | --- |
| Glycoside Hydrolase (GH) Families | GH16 | 7 |
|  | GH13 | 17 |
|  | GH11 | 1 |
|  | GH18 | 3 |
|  | GH19 | 3 |
|  | GH53 | 1 |
|  | GH76 | 1 |
|  | GH51 | 2 |
|  | GH73 | 5 |
|  | GH30 | 2 |
|  | GH32 | 9 |
|  | GH36 | 1 |
|  | GH101 | 2 |
|  | GH126 | 1 |
|  | GH105 | 2 |
|  | GH28 | 2 |
|  | GH23 | 10 |
|  | GH46 | 1 |
|  | GH26 | 1 |
|  | GH43 | 6 |
|  | GH68 | 1 |
|  | GH4 | 10 |
|  | GH5 | 1 |
|  | GH6 | 2 |
|  | GH0 | 13 |
|  | GH1 | 11 |
|  | GH2 | 3 |
|  | GH3 | 3 |
| Glycoside Transferase (GT) Families | GT26 | 3 |
|  | GT30 | 1 |
|  | GT31 | 1 |
|  | GT32 | 1 |
|  | GT20 | 1 |
|  | GT28 | 7 |
|  | GT0 | 6 |
|  | GT1 | 14 |
|  | GT2 | 48 |
|  | GT4 | 27 |
|  | GT13 | 3 |
|  | GT51 | 4 |
|  | GT8 | 1 |
| Polysaccharide Lyase (PL) Families | PL1 | 2 |
|  | PL9 | 1 |
| Carbohydrate Esterase (CE) Families | CE12 | 1 |
|  | CE14 | 7 |
|  | CE7 | 1 |
|  | CE6 | 2 |
|  | CE4 | 11 |
|  | CE9 | 4 |
| Carbohydrate-Binding Module (CBM) Families | CBM57 | 1 |
|  | CBM50 | 41 |
|  | CBM51 | 1 |
|  | CBM12 | 1 |
|  | CBM13 | 2 |
|  | CBM16 | 1 |
|  | CBM48 | 2 |
|  | CBM1 | 1 |
|  | CBM2 | 1 |
|  | CBM3 | 1 |
|  | CBM5 | 9 |
|  | CBM6 | 1 |
|  | CBM26 | 1 |

**References**

1. Hitch TC, Riedel T, Oren A, Overmann J, Lawley TD, Clavel TJIc. Automated analysis of genomic sequences facilitates high-throughput and comprehensive description of bacteria. 2021;1(1):16.
