# Supplementary material for: Genomic insights into the taxonomic status and bioactive gene cluster profiling of Bacillus velezensis RVMD2 isolated from desert rock varnish in Ma’an, Jordan
Source: PLoS One. 2025 Apr 24;20(4):e0319345. doi: 10.1371/journal.pone.0319345 (PMC12021177; doi:10.1371/journal.pone.0319345)
Supplement: S4 Table — (DOCX) [file pone.0319345.s004.docx]

**S4 Table.** Prophage regions identified in *Bacillus velezensis* strain RVMD2 genome using PHASTEST[1] Includes region length, completeness, score, total proteins, position, most common phage, and GC content.

| Region | Region Length | Completeness | Score | # Total Proteins | Region Position | Most Common Phage | GC % |
| --- | --- | --- | --- | --- | --- | --- | --- |
| 1 | 87.4Kb | incomplete | 60 | 119 | 390609-478057 | PHAGE_Bacill_SPbeta_NC_001884(52) | 36.69% |
| 2 | 64.5Kb | intact | 100 | 44 | 465550-530121 | PHAGE_Bacill_SPbeta_NC_001884(23) | 35.69% |
| 3 | 72.3Kb | intact | 150 | 71 | 624491-696797 | PHAGE_Bacill_phi105_NC_004167(27) | 45.33% |
| 4 | 40.5Kb | intact | 100 | 31 | 946714-987304 | PHAGE_Bacill_SPbeta_NC_001884(10) | 37.82% |
| 5 | 43.3Kb | intact | 150 | 63 | 289669-332984 | PHAGE_Bacill_SPP1_NC_004166(12) | 42.26% |
| 6 | 31.6Kb | intact | 120 | 41 | 381494-413141 | PHAGE_Brevib_Jimmer2_NC_041976(8) | 47.16% |
| 7 | 65Kb | intact | 130 | 76 | 125205-190288 | PHAGE_Bacill_vB_BtS_B83_NC_048762(8) | 43.20% |

**References**

1. Wishart DS, Han S, Saha S, Oler E, Peters H, Grant JR, et al. PHASTEST: faster than PHASTER, better than PHAST. Nucleic Acids Res. 2023;51(W1):W443-w50. Epub 2023/05/17. doi: 10.1093/nar/gkad382. PubMed PMID: 37194694; PubMed Central PMCID: PMCPMC10320120.
