# Supplementary figures and images for: Genomic insights into the taxonomic status and bioactive gene cluster profiling of Bacillus velezensis RVMD2 isolated from desert rock varnish in Ma’an, Jordan
Source: PLoS One. 2025 Apr 24;20(4):e0319345. doi: 10.1371/journal.pone.0319345 (PMC12021177; doi:10.1371/journal.pone.0319345)

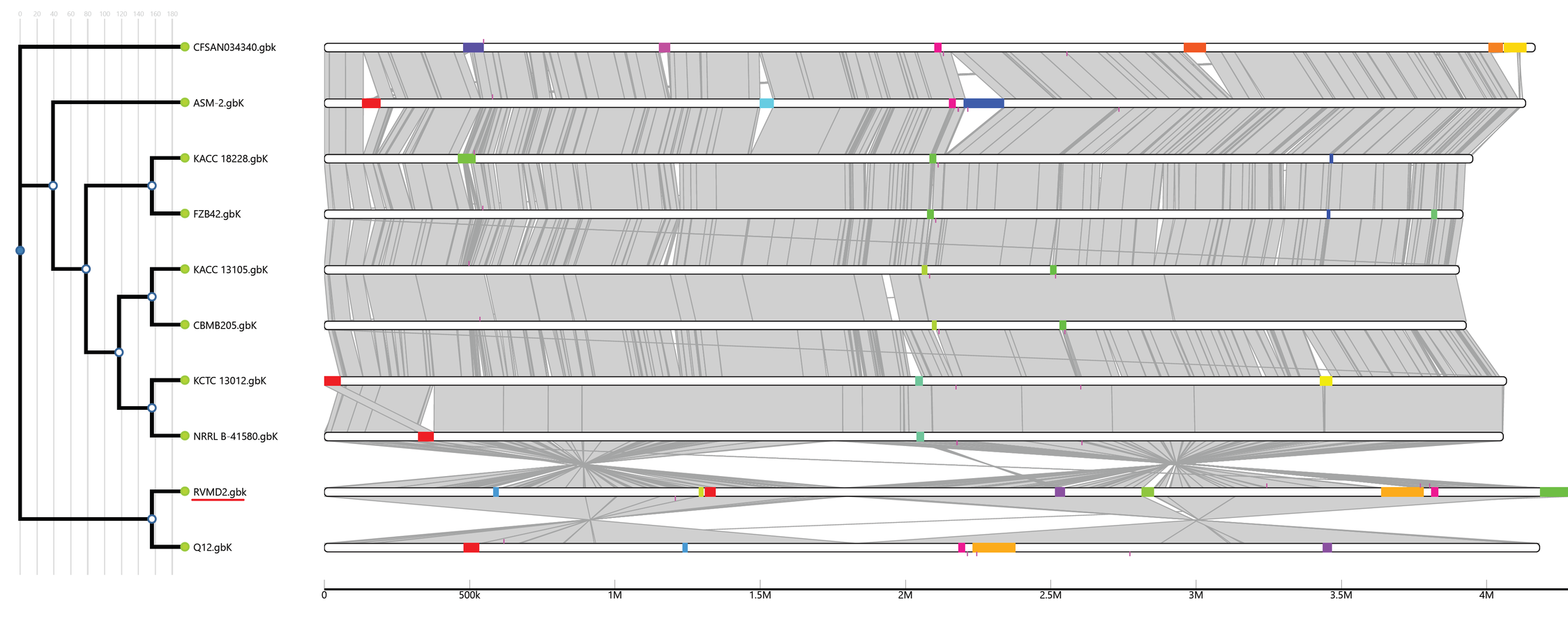

Supplement: S1 Fig — Genomic islands, representing probable horizontally acquired gene clusters, were identified and compared with close phylogenetic neighbors using IslandCompare (v1.0) https://islandcompare.ca/. This analysis distinguishes strain RVMD2 from its neighbors based on the number, type, and position of these islands. The left panel shows the phylogenetic tree, while the right panel displays synteny blocks and unique genomic islands, highlighting strain RVMD2. (TIF) [file pone.0319345.s005.tif]

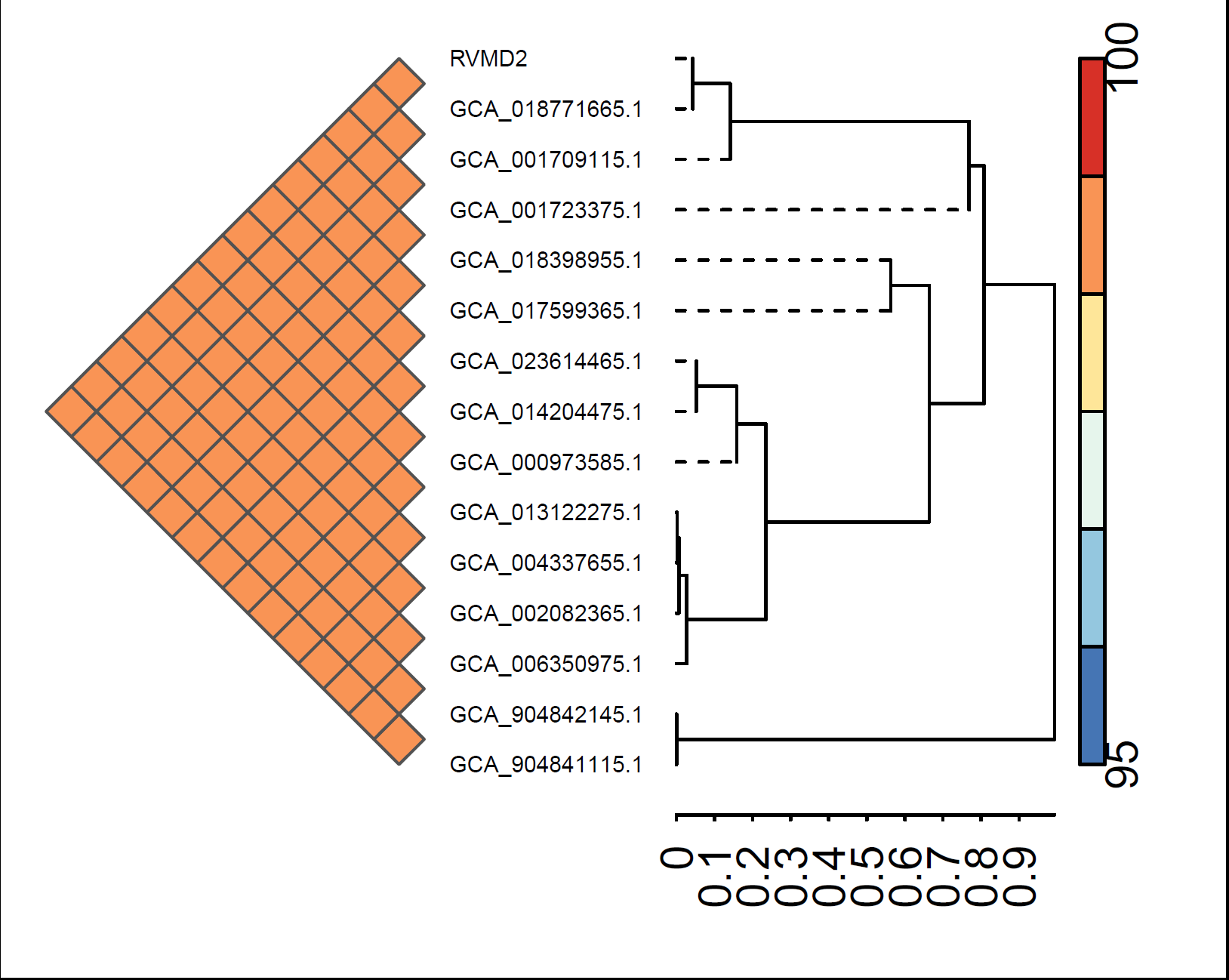

Supplement: S2 Fig — The analysis was performed using The Integrated Pan-Genome Analyser (IPGA) (https://nmdc.cn/ipga/), a web-based service. The heatmap illustrates the ANI values, with the color gradient representing the percentage identity, ranging from 95% to 100%. The dendrogram on the right shows the hierarchical clustering, indicating the evolutionary relationships among the strains. (TIF) [file pone.0319345.s006.tif]

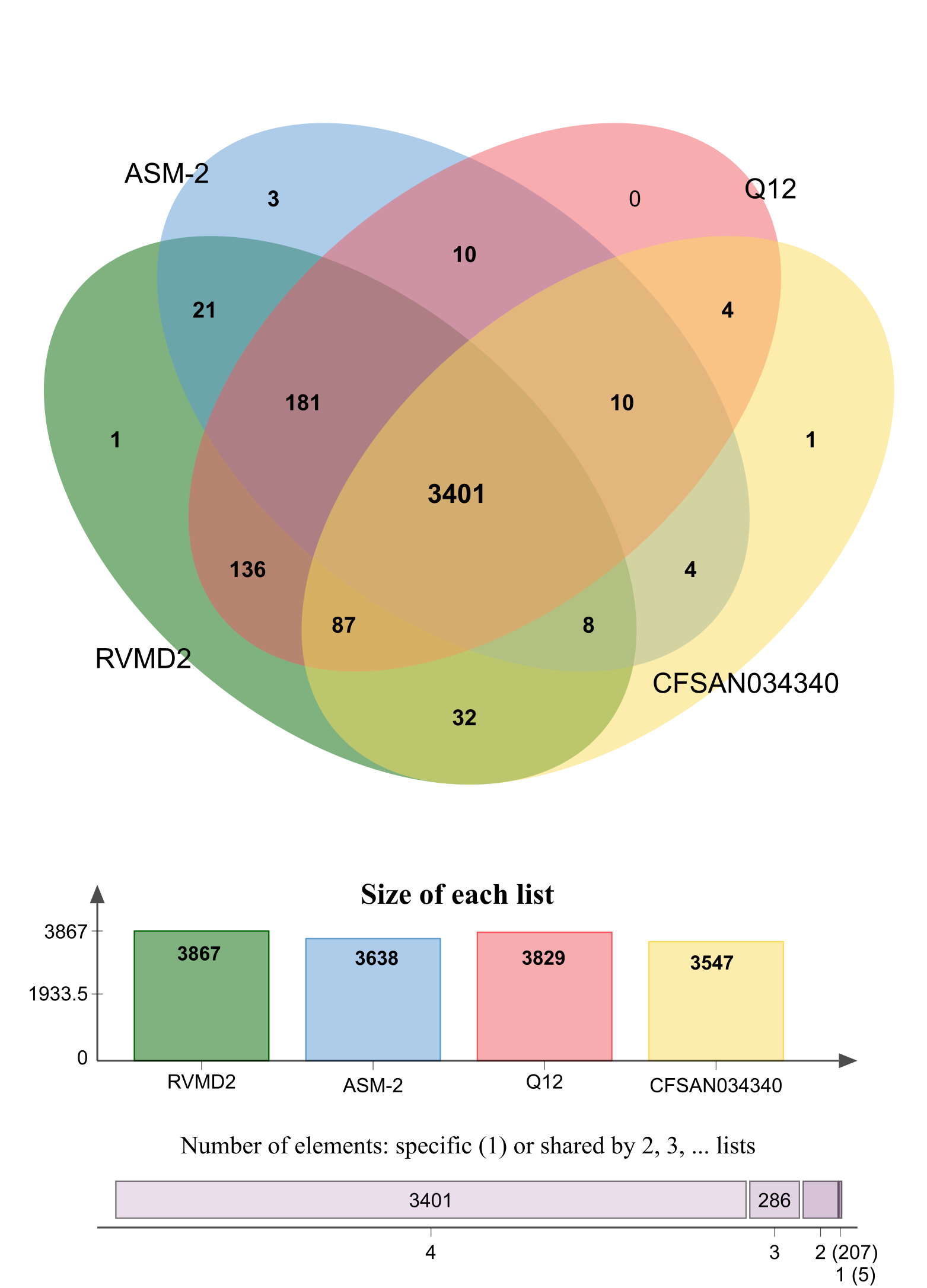

Supplement: S3 Fig — The overlapping regions indicate the number of gene families shared between the strains, while unique regions indicate gene families specific to each strain. The bar chart below the Venn diagram represents the size of each gene list for the strains. The bottom histogram shows the number of elements specific to one list or shared by two, three, or all four lists. (TIF) [file pone.0319345.s007.tif]
