# Supplementary material for: Genomic insights into the taxonomic status and bioactive gene cluster profiling of Bacillus velezensis RVMD2 isolated from desert rock varnish in Ma’an, Jordan
Source: PLoS One. 2025 Apr 24;20(4):e0319345. doi: 10.1371/journal.pone.0319345 (PMC12021177; doi:10.1371/journal.pone.0319345)
Supplement: Attachment — Submitted filename: Response to Reviewers.docx [file pone.0319345.s009.docx]

**Dr. Kamal Ahmad Qureshi, PhD**
Academic Editor
PLOS ONE

**Subject:** Response to Reviewer Comments for Manuscript PONE-D-24-46090

**Dear Dr. Qureshi,**

We thank you and the reviewers for the thorough evaluation of our manuscript titled **"Whole-Genome Sequencing and Comparative Genomics Reveal Prolific Bioactive Traits in *Bacillus velezensis* RVMD2 from Desert Rock Varnish in Ma'an, Jordan"** (Manuscript ID: PONE-D-24-46090). We have carefully addressed all the reviewers’ comments and the journal's requirements, and we have revised the manuscript accordingly.

In summary, we have made the following key updates:

1. **Journal Requirements:**
   - Included a statement in the Methods section clarifying that no permits were required for the fieldwork, as the site is publicly accessible, and no protected or endangered species were involved.
   - Revised the funding disclosure to clarify the funders’ role, stating explicitly: *"The funders had no role in study design, data collection and analysis, decision to publish, or preparation of the manuscript."*
   - We confirm that all data associated with this manuscript **are now freely available** without any restrictions. The data have been deposited in publicly accessible repositories, and the relevant accession numbers or identifiers (IDs) are provided within the manuscript to ensure full transparency and accessibility.
2. **Reviewer Comments:**
   - Addressed all major and minor reviewer comments in detail, including corrections to technical inaccuracies and enhancements to figure captions.
   - Clarified methodological details, such as phylogenetic analyses, ANI interpretations, and figure resolutions, to improve transparency and readability.

We have attached a detailed point-by-point response to all comments, highlighting how each concern was addressed in the revised manuscript.

We greatly appreciate the reviewers’ constructive feedback, which has helped us improve the clarity and rigor of our study. We are confident that the revised manuscript now meets the standards of PLOS ONE and respectfully submit it for further consideration.

Thank you for your time and attention. We look forward to your feedback.

Sincerely,

**Dr. Al-Awaida**

**Reviewer #1:**

a) **Problem Statement: The manuscript lacks a clear and compelling problem statement. While it emphasizes the extremophilic origin of *Bacillus velezensis* RVMD2, it does not adequately frame the scientific gap or justify the novelty of isolating this strain. Can you introduce a specific research problem, such as the underexplored bioactive potential of microorganisms from desert varnish, and align it with broader biotechnological applications viewer?**
b) **Objectives: The study outlines various analyses (e.g., genomic, phylogenetic, bioactive compound identification) but does not explicitly state clear, measurable objectives. Please clarify which to achieve?**

**To determine the genomic adaptations of *B. velezensis* RVMD2 to extreme environments or ANTIMICROBIAL or BIOREMEDIATION?**

**Our response:** To address these concerns, we have revised the manuscript to provide a clear research gap and align the study with broader biotechnological applications. Additionally, we have explicitly stated the objectives to ensure they are measurable and directly aligned with the study's goals. In the Revised Manuscript with Track Changes (Line 92), we have included the following updated text:

- *To the best of our knowledge, no studies have comprehensively investigated the bioactive gene potential of microorganisms thriving in this unique environment. Desert varnish, an underexplored habitat, may harbor microbial communities with novel genes and secondary metabolites that could have significant applications in medicine and agriculture. Recognizing the need to explore this potential, this study focuses on Bacillus velezensis strain RVMD2, isolated from rock varnish in the Ma'an Desert, Jordan.*
- *This study aims to determine the taxonomic identity and provide a comprehensive characterization of the phylogenetic, genomic, and taxonomic features of B. velezensis RVMD2. Specifically, we seek to establish its taxonomic placement through phylogenomic analysis and investigate genes related to antibiosis, secondary metabolite biosynthesis, and other distinctive traits. These features are compared with those from the broader Bacillus genus to assess its potential contributions to biotechnological applications. To achieve these goals, we employ a multiphase classification strategy that integrates whole-genome shotgun sequencing with rRNA gene amplicon analysis, providing a robust framework for understanding the strain's genomic potential*.

c) **Methods and Environmental Relevance: There is an inconsistency in claiming the extremophile nature of the strain versus its cultivation at 37°C in a controlled laboratory setting. This raises concerns about how well the findings translate to its natural habitat.**

**Our response:** We would like to clarify that we do not claim *Bacillus velezensis* RVMD2 itself is an extremophile. Instead, we emphasize that it was isolated from an extreme environment—desert rock varnish—which is characterized by harsh conditions such as high temperatures, UV exposure, and limited nutrients. The occurrence of non-extremophilic microorganisms in extreme environments is well-documented, as such organisms often possess specific adaptive features that enable them to survive under these challenging conditions. We believe that *B. velezensis* RVMD2 may exhibit similar adaptations, and our study aims to explore its genomic features that could contribute to its survival in such an environment**.**

d) **Strength of Claims: The manuscript suggests the presence of novel antimicrobial compounds and bioactive traits BUT lacks experimental validation or biochemical characterization to substantiate these claims, can you couple your claim with proof on antimicrobial potential through wet-lab experiments.**

**Our response:** As stated in the limitations section of our manuscript, the bioactive potential identified in the genome requires further experimental validation in the laboratory. This will be the focus of our upcoming project, where we plan to test *B. velezensis* RVMD2 for its bioactive metabolites and employ techniques such as HPLC, mass spectrometry, and antimicrobial assays to fully characterize these metabolites and confirm their novelty. We believe that the work presented here provides a strong foundation and represents a crucial first step in understanding the genomic potential of this strain. By identifying biosynthetic gene clusters (BGCs) and highlighting the strain’s biosynthetic capabilities, this study sets the stage for future investigations into its biochemical properties and practical applications.

e) **Genomic and Comparative Analysis: The comparative analysis is detailed but focuses heavily on the technical aspects without adequately linking findings to practical implications.**
f) **The manuscript provides a robust genomic dataset but falls short in demonstrating its relevance and potential applications.**

**Our response:** As highlighted before, the current study primarily focuses on characterizing the genomic features of *Bacillus velezensis* RVMD2, including its biosynthetic gene clusters (BGCs) and adaptive traits. While we have provided a robust genomic dataset, we acknowledge the need to more explicitly highlight its relevance and potential applications. This will be a central focus of our upcoming project, where we plan to test *B. velezensis* RVMD2 for its bioactive metabolites and employ techniques such as HPLC, mass spectrometry, and antimicrobial assays to fully characterize these metabolites and confirm their novelty. These experiments will allow us to explore the strain’s potential applications in medicine, agriculture, and other biotechnological fields**.**

**Reviewer #2:**

- **Line 20: Comment: Italicize *B. velezensis*.**
  **Our response:** This correction has been implemented in the manuscript.
- **Line 22:Comment:** **Italicize the genus name in *B. velezensis***.
  **Our response:** The genus name has been italicized as requested throughout the manuscript.
- **Line 39: Comment: "rock varnish—a thin" has an extra space; remove it.
  Our response:** The extra space has been removed for consistency and readability.
- **Line 40–42: Our response: Some more recent literature on whole-genome sequencing and genome mining of microbes from extreme environments, such as deserts, should be cited. For example: Fatima et al., 2024,** [**https://doi.org/10.1016/j.genrep.2024.102050**](https://doi.org/10.1016/j.genrep.2024.102050)**.**
  **Our response:** Thank you for the suggestion. The following text has been added to Line 44 in the revised manuscript: *"Genome mining of desert-isolated strains has enabled the identification of biosynthetic gene clusters (BGCs) linked to the production of diverse bioactive compounds, including antibiotics (4). These findings underscore the potential of extreme environment-derived microorganisms as promising sources for novel antibiotic discovery."*
- **Line 46: Comment: Italicize *Bacillus*. This error occurs frequently in the manuscript; check and correct all scientific names.**
  **Our response:** All scientific names, including *Bacillus*, have been reviewed and italicized consistently throughout the manuscript.
- **Lines 57–61: Comment:** Why are all these lines italicized? Check and update.
  **Our response:**  The formatting issue has been reviewed, and unnecessary italicization has been corrected.
- **Line 77: Comment: Italicize *Botryosphaeria dothidea*.**
  **Our response:** This correction has been implemented.
- **Line 98: Comment:** **Check the coordinates; ensure the degree sign (°) is present.**
  **Our response:**  The coordinates have been reviewed, and the degree sign (°) has been added where necessary.
- **Figures: Comment:** **The quality of most figures is acceptable, but the resolution of the circular genome and figures such as Fig. 1, Fig. 5, Fig. 7, and Fig. 8 should be improved for clarity.**
  **Our response:** The resolution of the mentioned figures has been improved to ensure clarity and compliance with publication standards.
- **AntiSMASH Output: Comment: The methodology mentions analyzing WGS data with AntiSMASH, but the AntiSMASH output table is missing from the results. Include the table in the results section.**
  **Our response:** We thank the reviewer for their observation. The table detailing gene clusters involved in secondary metabolite synthesis, previously included as Table S5 in the supplementary files, has now been moved to the main text of the manuscript for better visibility, please check Table 6. This table complements the visualization of secondary metabolite gene clusters presented in Figure 8. We hope this adjustment addresses the reviewer’s concern and enhances the clarity of our results.

**Reviewer #3:**

- **The genome was sequenced, resulting in 4,212,579 bp, but the final structure of the chromosome was not obtained. The assembly comprises 112 contigs, making chromosomal localization or comparisons with other chromosomes problematic. For example, the observed inversions may be artifacts**.

**Our response:** We thank the reviewer for their insightful comments regarding the genome assembly and its implications for chromosomal localization and structural comparisons. We acknowledge that the Bacillus velezensis RVMD2 genome assembly consists of **112 contigs**, which limits the precise identification of structural rearrangements and genomic organization. However, we would like to clarify that the tools used in this study—such as **Proksee, IslandViewer 4, PHASTEST, and FastANI**—are specifically designed to handle fragmented genomes and remain reliable for visualizing genomic features and calculating average nucleotide identity (ANI).

For example:

- **Figure 2** (circular genome map) illustrates genomic features such as GC content, GC skew, and open reading frames (ORFs), providing an overview of the strain’s genomic structure.
- **Figure 7** highlights genomic islands and prophage regions predicted using IslandViewer 4 and PHASTEST, respectively. These predictions are based on sequence composition and annotations and are considered reliable despite the fragmented assembly.
- **Figure 10** demonstrates conserved genomic regions and alignments with closely related strains, supported by high ANI values (99.99%, 99.85%, and 99.18% with strains Q12, CFSAN034340, and ASM-2, respectively), which validate the evolutionary relationships and functional similarities.

While the fragmented nature of the assembly may introduce some limitations, such as potential artifacts in structural comparisons (e.g., inversions), the results align with standard practices in bacterial genomics and provide meaningful insights into the genomic potential of B. velezensis RVMD2.

To address these points, we have updated the manuscript as follows:

1. **Added clarification in the results section (Lines 518-522)**:
   "The genomic islands were identified using IslandViewer 4 [32], and the prophage regions were predicted using PHASTEST [34]. These predictions are based on sequence composition and annotations, and while the level of fragmentation in the genome assembly may have some influence, the results are still considered reliable and align with standard practices in bacterial genomics."
2. **Updated the limitations section (Lines 688-702):**:
   "The fragmented nature of the Bacillus velezensis RVMD2 genome assembly also limits the ability to precisely determine structural rearrangements and genomic organization. Nevertheless, the analyses and tools employed align with standard practices and provide meaningful insights into the strain’s genomic potential."

**Reviewer #4**

- **L193: Confirm whether datasets are currently under embargo.**
  **Our response:** All data mentioned in this manuscript, including the Whole Genome Shotgun (WGS) project, raw FASTQ files from genome sequencing, and Sequence Read Archive (SRA) data, are now publicly available without any restrictions. The relevant accession numbers or identifiers (IDs) are provided within the manuscript to ensure full transparency and accessibility.
- **L239: What are the distance units? Please indicate.**

**Our response:** The scale bar represents a genetic distance of 0.01 substitutions per site. The figure caption has been updated in the revised manuscript to include this information for clarity.

- **L375: "ANI coverage of 91.1648%, which is higher than the ANI threshold cut-off value (95–96%)". Rephrase this statement for clarity, as 91 < 95.**
  **Our response:** Thank you for pointing out this inconsistency. Upon review, we realized that the text mistakenly referred to the ANI coverage (91.1648%) instead of the ANI percentage (97.803%), which is indeed above the species threshold cut-off value of 95–96%. The corrected text now reads:

"Based on Table 4, the analysis using the EzBiome Genome-ID tool identified Bacillus velezensis as the top match for bacterial isolate RVMD2, with an ANI of 97.803%, 16S similarity of 99.92%, recA identity of 98.563%, rplC identity of 99.524%, Mash identity of 97.8%, and ANI coverage of 91.1648%. The ANI percentage (97.803%) is higher than the species demarcation threshold of 95–96% (44)."

- **L401: The phylogenetic tree places *B. velezensis* strain RVMD2 in a well-supported clade with high bootstrap values. However, the closest clade contains three different species, suggesting that more species/strains should be included to improve resolution. Agree?**
  **Our response:** The phylogenetic tree was constructed using the TYGS (Type Strain Genome Server) platform, which automatically selects related species based on the query genome (B. *velezensis* RVMD2). We did not manually curate the species included in the analysis, as TYGS determines the most relevant genomes for comparison.

All genomes within the clade containing our bacterial strain are part of the same species cluster. This aligns with the reclassification of B. methylotrophicus KACC 13105 and B. amyloliquefaciens subsp. plantarum FZB42 as synonyms of B. velezensis NRRL B-41580, as established by Dunlap et al. (2016). Consequently, we designate our strain as B. velezensis RVMD2, consistent with this taxonomic framework.

- **L408: Explain how the *Bacillus* species included in the study were selected.**
  **Our response:** The Bacillus species included in the study were determined using the TYGS platform, which identifies the most relevant related genomes based on the query genome. TYGS selects and incorporates genomes from curated databases to ensure accurate phylogenetic and taxonomic comparisons. We did not manually include additional species, as this approach ensures objectivity and consistency in identifying closely related species for analysis.
- **L518: In the version available for review, it is impossible to read the content of this figure. Ensure the resolution is sufficient for publication.**
  **Our response:** We appreciate the reviewer’s feedback. The figure resolution has been improved in the revised manuscript to ensure clarity and readability, making it suitable for publication.
- **L635: The manuscript discusses genomic findings, but how meaningful are these statements given the number of contigs in the assembly? Please clarify.**
  **Our response:** We acknowledge the potential limitations introduced by the fragmented genome assembly of B. velezensis RVMD2. While the assembly consists of multiple contigs, we used tools such as FastANI that are designed to provide robust ANI estimates and meaningful insights even for draft genomes.
- FastANI calculates ANI using high-quality alignments of conserved genomic regions, ensuring reliability despite genome fragmentation. The high ANI values (99.99%, 99.85%, and 99.18% for Q12, CFSAN034340, and ASM-2, respectively) indicate strong genomic similarity, supporting the evolutionary relationships and functional similarities of RVMD2 with its closest strains.
- We note that this concern about the fragmented genome was also raised by another reviewer. In response, we have transparently discussed this limitation in the manuscript and emphasized how the use of specialized tools like FastANI, designed for fragmented assemblies, supports the robustness of our conclusions.
- Additionally, while Figure 10 may not perfectly represent the genomic organization due to potential contig breaks, the reciprocal mappings shown in the figure demonstrate a high level of sequence conservation, reinforcing the validity of our results. This limitation has been explicitly addressed in the revised manuscript.
- **L57: Please remove italics after species name.**
  **Our response:** The unnecessary italics after the species name have been removed in the revised manuscript.
- **L139: Replace . with ,.**
  **Our response:** The period has been replaced with a comma, as suggested.
- **L154: Add a space before "web please".**
  **Our response:** A space has been added before "web please" to correct the formatting issue.
- **L395: "we designating it" please change to "designated".**
  **Our response:** The phrase "we designating it" has been corrected to "designated."
- **L719: Please add journal name and complete citation if needed.**
  **Our response:** The journal name and complete citation have been added. Furthermore, all references in the manuscript have been thoroughly reviewed to ensure they are complete, accurate, and properly formatted
