# Supplementary material for: Genomic insights into the taxonomic status and bioactive gene cluster profiling of Bacillus velezensis RVMD2 isolated from desert rock varnish in Ma’an, Jordan
Source: PLoS One. 2025 Apr 24;20(4):e0319345. doi: 10.1371/journal.pone.0319345 (PMC12021177; doi:10.1371/journal.pone.0319345)
Supplement: Attachment — Submitted filename: Response_to_Reviewers_auresp_2.docx [file pone.0319345.s010.docx]

**Response to Reviewer #1:**
Thank you for your feedback. We have carefully revised the title to ensure it accurately reflects the study’s objectives. The new title, **"Genomic insights into the taxonomic status and bioactive gene cluster profiling of Bacillus velezensis RVMD2 isolated from desert rock varnish in Ma'an, Jordan"** explicitly highlights the key research components, including taxonomic classification, genomic characterization, and secondary metabolite gene analysis. We believe this revision aligns well with the study’s scope and objectives.
